# A Case of Delayed Refractory *Mycobacterium mageritense* Abdominal Wall Abscess in a Kidney Transplant Recipient

**DOI:** 10.1002/iju5.70042

**Published:** 2025-05-09

**Authors:** Hisashi Sakurai, Teppei Okamoto, Tomoko Hamaya, Hirotake Kodama, Naoki Fujita, Hayato Yamamoto, Kazuyuki Mori, Takeshi Fujita, Atushi Imai, Reiichi Murakami, Hirofumi Tomita, Shingo Hatakeyama

**Affiliations:** ^1^ Department of Urology Hirosaki University School of Medicine Aomori Japan; ^2^ Department of Cardiology and Nephrology Hirosaki University School of Medicine Aomori Japan

**Keywords:** abdominal wall abscess, kidney transplant recipients, *Mycobacterium mageritense*, non‐tuberculous mycobacterium

## Abstract

**Introduction:**

*Mycobacterium mageritense* ( *M. mageritense* ), a rare non‐tuberculous mycobacterium (NTM), can cause infections in immunocompromised patients, including kidney transplant recipients. We present a case of an abdominal wall abscess caused by *M. mageritense* following a living donor kidney transplant.

**Case Presentation:**

A 58‐year‐old woman, post‐ABO‐incompatible kidney transplant, developed an abscess at the site of a removed peritoneal dialysis catheter. Initial antibiotics were ineffective, and pus cultures identified *M. mageritense* . Surgical drainage and levofloxacin‐linezolid therapy controlled the infection temporarily. Despite clinical improvement, the abscess recurred 30 days post‐discharge, which required repeated antibiotic use and adjustments to immunosuppression. Reducing mycophenolate mofetil while maintaining tacrolimus stabilized the infection, and prophylactic levofloxacin was continued post‐discharge to prevent relapse.

**Conclusion:**

Effective infection control requires careful immunosuppressive adjustment and long‐term antibiotic use to balance graft preservation with infection risk.

**Summary**

- This case highlights the difficulty of managing rare infections like *Mycobacterium mageritense* in kidney transplant recipients.
- Careful immunosuppressive adjustment and prolonged antibiotics are key to balancing infection control and graft preservation.

## 1 Introduction

Non‐tuberculous mycobacteria (NTM) infections are rare and pose significant challenges in immunosuppressed patients [1], particularly following kidney transplantation [2, 3]. Among these, *Mycobacterium mageritense* ( *M. mageritense* ), a rapidly growing mycobacterium (RGM), has been reported in association with catheter‐related infections in patients with end stage kidney disease [4]. We present a case of a delayed refractory *M. mageritense* abscess at the site of a removed PD catheter in a kidney transplant recipient.

## 2 Case Presentation

A 58‐year‐old woman with IgA nephropathy underwent an ABO‐incompatible living‐donor kidney transplant from her sister. A peritoneal dialysis (PD) catheter was removed during the same procedure. Induction immunosuppressive therapy included tacrolimus, mycophenolate mofetil (MMF), and prednisone, along with perioperative rituximab and basiliximab. She was discharged from the hospital without any surgical site infections.

**FIGURE 1:**
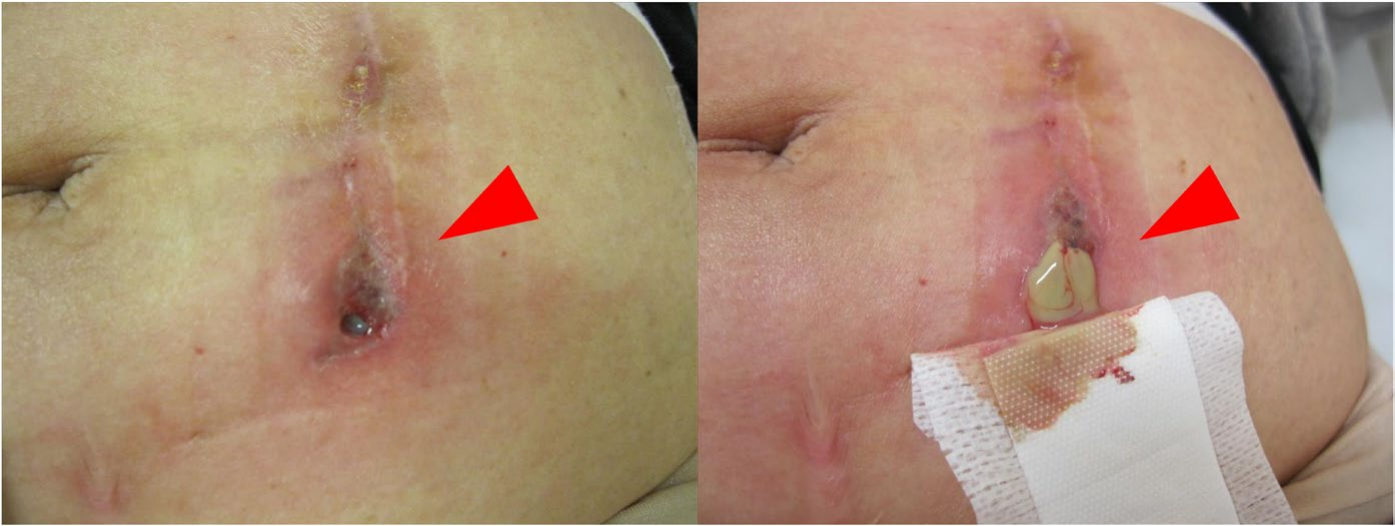
On postoperative day 55, we observed redness, pain, and bloody pus discharge at the PD catheter removal site.

**FIGURE 2:**
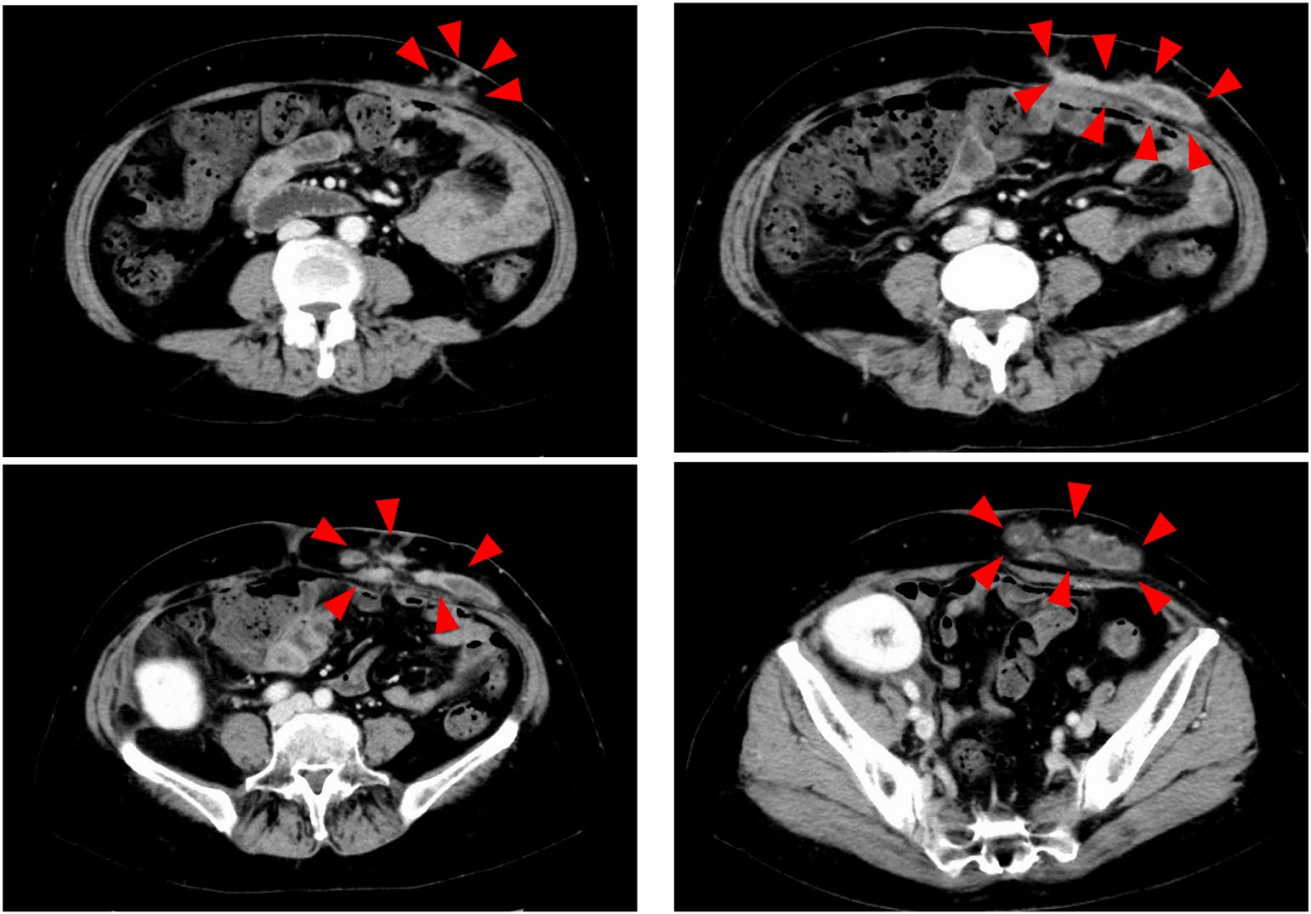
A CT scan revealed a widespread abscess at the site of the PD catheter removal.

**FIGURE 3:**
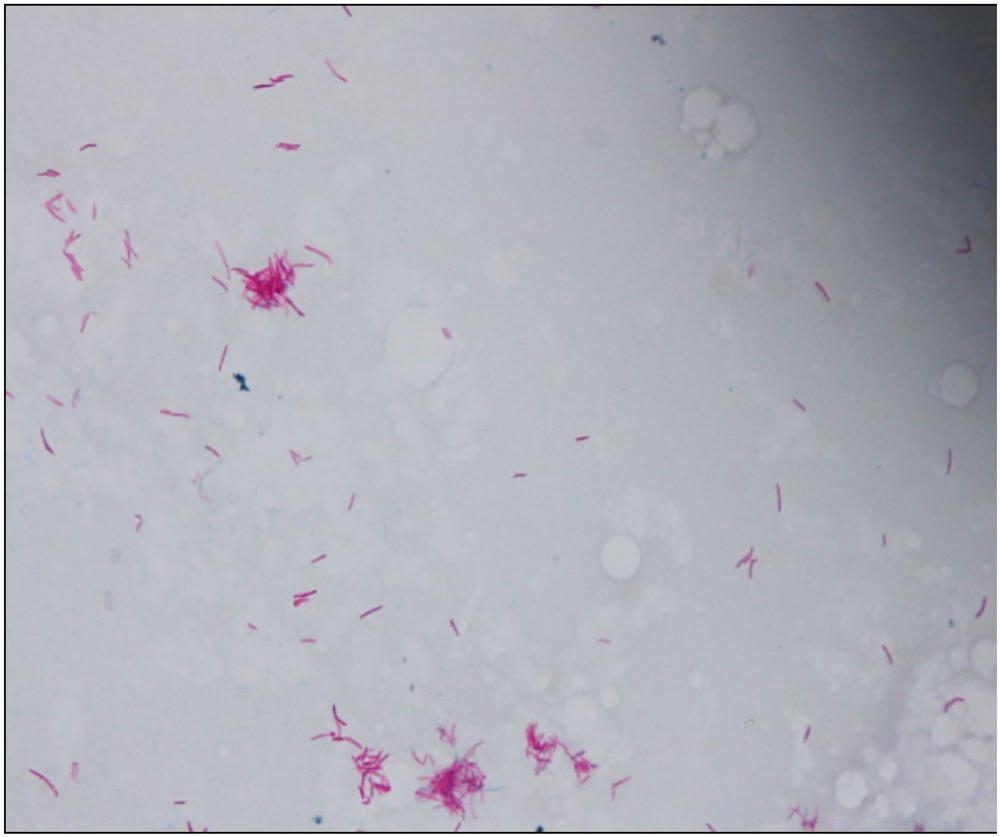
*Mycobacterium mageritense* was detected in the pus culture using a Ziehl‐Neelsen stain.

**FIGURE 4:**
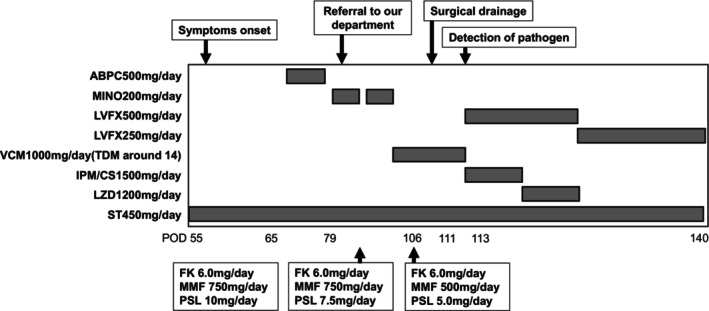
Antimicrobial agents used and their dosing, along with the changes in immunosuppressive drug doses following the onset of abdominal wall abscess.

Fifty‐five days after surgery, she developed bloody exudate and pain at the site of the removed PD catheter (Figure 1). Laboratory studies showed no signs of systemic inflammation, and both initial pus cultures and Gram staining were negative for bacteria. Despite treatment with ampicillin and frequent pus drainage, her symptoms did not improve, and she was referred to our department. A Computed tomography revealed two large subcutaneous abscesses extending to the rectus abdominis fascia at the catheter removal site (Figure 2). The patient underwent extensive debridement of fat tissues around the abscess under general anesthesia. We considered the possibility of an infection caused by methicillin‐resistant *Staphylococcus aureus* ; therefore, we initiated treatment with vancomycin. Postoperative cultures and PCR identified *M. mageritense* (Figure 3). Once sensitivities were confirmed, she was treated with linezolid and levofloxacin, while MMF and PSL were carefully tapered to balance infection control and rejection risk. The changes in immunosuppressive drugs and antimicrobial agents are summarized in Figure 4. Her symptoms fully resolved 21 days after starting treatment and had no sign of subcutaneous abscess, and she was discharged without prolonged antibiotic therapy.

Despite initial clinical improvement, the abscess recurred 30 days after discharge, necessitating readmission for pus drainage. Although *M. mageritense* was not detected in the pus culture, antibiotic therapy with linezolid and levofloxacin was reinitiated. The patient showed clinical improvement and continued treatment with levofloxacin and linezolid for 2 months to prevent further recurrence. However, severe pancytopenia, likely caused by linezolid, necessitated its discontinuation. The patient continued levofloxacin alone for an additional month. Six months after the cessation of antibiotic therapy, the patient remained free of recurrent infection.

## 3 Discussion

*M. mageritense* infections are rare, even in transplant recipients [3]. This case highlights the challenges in managing such infections in immunocompromised patients, where balancing the need for infection control with the risk of graft rejection is critical.

*M. mageritense* is an NTM that is classified as rapidly growing mycobacteria (RGM) and was first reported as a new species in late 1990's [5]. It is commonly found in environments like soil, organic materials, and treated water sources [6]. M. *mageritens* is known for causing skin and soft tissue infections, particularly in individuals with catheters or implants [7, 8]. Diagnosing NTM infection is challenging when using Gram staining. NTM are stained weakly as gram‐positive bacilli. However, they may sometimes fail to be stained as either gram‐positive or gram‐negative, resembling scratches on glass [9]. In this case, no microorganisms were detected in the initial Gram staining. However, a large amount of pus and infected subcutaneous tissue was obtained during surgical debridement, which provided sufficient material for detecting microorganisms such as NTMs. Ziehl‐Neelsen staining was subsequently performed, indicating the involvement of mycobacteria. In similar cases, where bacterial organisms are not identified through Gram staining, it is essential to actively pursue mycobacterial staining. The biochemical characteristics of mycobacteria provide clues for species differentiation, but identifying the species based solely on these characteristics is not possible. Therefore, we identified this species using loop‐mediated isothermal amplification PCR method. In this case, the patient developed a delayed abscess at the PD catheter removal site, which is an unusual course of bacterial infection even in kidney transplant recipients. While NTM infections are rare in transplant patients, Masalmani M. Al et al. reported several case series of kidney transplant recipients who developed NTM infections [3]. The period from surgery to the onset of infection ranged from one to 4 months, which was similar to the course in this case. We should consider the possibility of NTM infection in cases of delayed abscess formation around surgical wounds.

RGM, including *M. mageritense* , exhibit varying susceptibility to antibiotics [5, 10]. In this case, the pathogen was sensitive to amikacin, sulfamethoxazole‐trimethoprim, levofloxacin, and linezolid but resistant to clarithromycin, a common pattern in RGMs. The patient had already been receiving sulfamethoxazole‐trimethoprim prophylaxis for pneumocystis pneumonia prior to the onset of the abdominal wall abscess. Furthermore, the use of amikacin requires careful monitoring of blood concentrations to prevent severe kidney dysfunction. A standardized treatment protocol has not been defined; however, previous studies have indicated that *M. mageritense* is susceptible to specific combinations of antibiotics [8, 11]. Therefore, in this case, we adopted a combination therapy of levofloxacin and linezolid. The biggest issue is that we discontinued antibiotic treatment immediately after the patient recovered from the abscess. Since her infectious wounds appeared to have healed, we assumed that continuing antibiotics was unnecessary. Prolonged therapy is often recommended to prevent recurrence, typically lasting three to 6 months in transplant recipients [3].

One of the challenging aspects of this case was the management of immunosuppression. No PD catheter‐related infections were observed during PD. We speculate that *M. mageritense* colonized the site during PD, remaining asymptomatic until post‐transplant immunosuppression allowed progression to infection. In addition, balancing the reduction of immunosuppressive agents to aid in infection control with the risk of graft rejection was critical. Initially, MMF was tapered while maintaining tacrolimus at a stable dose. MMF primarily inhibits the proliferation of T and B lymphocytes, which are essential for mounting an effective immune response against infections. In line with a previous study [12], reducing MMF may be a reasonable approach to fighting against the infection. There are no standardized protocols for tapering immunosuppression in such infections.

In this case, adjustments were made based on clinical experience and through consultation with nephrologists to ensure safety.

The absence of prior PD‐related infection made this abdominal wall abscess difficult to predict. The PD catheter had been placed in the left abdomen, opposite the transplanted kidney. Since the catheter lies in the peritoneal cavity and the graft in the retroperitoneum, placement usually poses no problem. However, post‐transplant infection at the exit site may require surgical drainage. Therefore, placing the catheter in the left lower quadrant may be preferable, maintaining distance from the graft to reduce infection risk. In patients scheduled to undergo kidney transplantation, placing the PD catheter in the left lower quadrant may be preferable.

## 4 Conclusion

This case illustrates the complexity of managing *M. mageritense* infections in kidney transplant recipients. Careful and detailed treatment plans are essential to control the infection while preserving graft function.

## Ethics Statement

This research was conducted in accordance with the provisions of the Declaration of Helsinki.

## Consent

We obtained consent from the patient's parents for the publication of this case report.

## Conflicts of Interest

The authors declare no conflicts of interest.
